# The role of type VI secretion system genes in antibiotic resistance and virulence in *Acinetobacter baumannii* clinical isolates

**DOI:** 10.3389/fcimb.2024.1297818

**Published:** 2024-02-07

**Authors:** Pu Li, Sirui Zhang, Jingdan Wang, Mona Mohamed Al-Shamiri, Kai Luo, Shuyan Liu, Peng Mi, Xiaokang Wu, Haiping Liu, Huohuan Tian, Bei Han, Jin’e Lei, Shaoshan Han, Lei Han

**Affiliations:** ^1^ School of Public Health, Xi’an Jiaotong University Health Science Center, Xi’an, Shaanxi, China; ^2^ Department of Microbiology and Immunology, School of Basic Medical Sciences, Xi’an Jiaotong University Health Science Center, Xi’an, Shaanxi, China; ^3^ Department of Laboratory Medicine, Shaanxi Provincial People’s Hospital, Xi’an, China; ^4^ Department of Laboratory Medicine, the Second Affiliated Hospital of Xi’an Jiaotong University, Xi’an, China; ^5^ Department of Laboratory Medicine, Xi’an Daxing Hospital, Xi’an, China; ^6^ Department of Respiratory and Critical Care Medicine, West China Hospital, Sichuan University, Chengdu, China; ^7^ Department of Laboratory Medicine, the First Affiliated Hospital of Xi’an Jiaotong University, Xi’an, China; ^8^ Department of Hepatobiliary Surgery, the First Affiliated Hospital of Xi’an Jiaotong University, Xi’an, China

**Keywords:** *Acinetobacter baumannii*, T6SS, TssB, TssD, TSSM, antimicrobial resistance, virulence

## Abstract

**Introduction:**

The type VI secretion system (T6SS) is a crucial virulence factor in the nosocomial pathogen *Acinetobacter baumannii*. However, its association with drug resistance is less well known. Notably, the roles that different T6SS components play in the process of antimicrobial resistance, as well as in virulence, have not been systematically revealed.

**Methods:**

The importance of three representative T6SS core genes involved in the drug resistance and virulence of *A. baumannii*, namely, *tssB*, *tssD* (*hcp*), and *tssM* was elucidated.

**Results:**

A higher ratio of the three core genes was detected in drug-resistant strains than in susceptible strains among our 114 *A. baumannii* clinical isolates. Upon deletion of *tssB* in AB795639, increased antimicrobial resistance to cefuroxime and ceftriaxone was observed, alongside reduced resistance to gentamicin. The Δ*tssD* mutant showed decreased resistance to ciprofloxacin, norfloxacin, ofloxacin, tetracycline, and doxycycline, but increased resistance to tobramycin and streptomycin. The *tssM*-lacking mutant showed an increased sensitivity to ofloxacin, polymyxin B, and furazolidone. In addition, a significant reduction in biofilm formation was observed only with the Δ*tssM* mutant. Moreover, the Δ*tssM* strain, followed by the Δ*tssD* mutant, showed decreased survival in human serum, with attenuated competition with *Escherichia coli* and impaired lethality in *Galleria mellonella*.

**Discussion:**

The above results suggest that T6SS plays an important role, participating in the antibiotic resistance of *A. baumannii*, especially in terms of intrinsic resistance. Meanwhile, *tssM* and *tssD* contribute to bacterial virulence to a greater degree, with tssM being associated with greater importance.

## 1 Introduction

*Acinetobacter baumannii*, an opportunistic nosocomial pathogen, shows an extraordinary ability to acquire resistance to antimicrobials (AlAmri et al., 2020). An increasing number of multidrug-resistant (MDR), extremely-drug-resistant (XDR), and even pan-drug-resistant (PDR) strains impose a great burden on modern medicine (Ramirez et al., 2020). Aa a member of ESKAPE (*Enterococcus faecium*, *Staphylococcus aureus*, *Klebsiella pneumonia*, *Acinetobacter baumannii*, *Pseudomonas aeruginosa*, and *Enterobacter*) (Pogue et al., 2015), *A. baumannii* poses a great challenge in hospital-acquired infections, such as ventilator-associated pneumonia, bloodstream infections, surgical site or wound infections, urinary tract infections, and meningitis. It is particularly problematic for immunocompromised and debilitated patients confined to intensive care units (ICUs) (McConnell et al., 2013; Wong et al., 2017).

Different antibiotic-resistance mechanisms have been identified in *A. baumannii*. Moreover, adaptive resistance plays a key role in facilitating the rapid response of bacteria to antibiotic challenges and external stresses (Arzanlou et al., 2017; Christaki et al., 2020). This can result in decreased antimicrobial susceptibility by altering the expression of porins and efflux pumps (Fernández and Hancock, 2012). Similar to antibiotic resistance, *A. baumannii* can adapt to stresses such as desiccation and oxidation, which are dependent on its specific structure (Kwon et al., 2017). However, we have a limited understanding of the basic biological processes that drive *A. baumannii* to successfully infiltrate the healthcare environment, adapt to antibiotics and environmental stresses, and cause diseases. A deep understanding of this process may provide novel potential targets for the development of future antimicrobials and vaccines.

Protein secretion systems are critical virulence structures in *A. baumannii* and have received increased attention recently (Skariyachan et al., 2019). They can transport virulence factors into the surrounding environment or directly into neighboring cells (Gerlach and Hensel, 2007). Recent studies have shown that secretion systems are involved in the antibiotic resistance of *A. baumannii* (Elhosseiny et al., 2019). The type VI secretion system (T6SS) is the most frequently reported secretion system and is important for microbial communication within the environment and hosts; thus, it may contribute to antibiotic adaptation and drug resistance.

T6SS is a vital dynamic protein delivery apparatus that is analogous to the tail assembly of contractile bacteriophages that can inject toxic effector molecules into both prokaryotic and eukaryotic prey cells (Alcoforado Diniz et al., 2015). It is composed of a membrane complex, cytoplasmic baseplate, and contractile tail tube/sheath complex encoded by 13 well-conserved core genes (Nguyen et al., 2018). TssD (Hcp) is an important protein in the tail tube/sheath complex and is commonly used as a marker of T6SS activity (Weber et al., 2017). Hexameric TssD forms a tube-like structure arranged with helical symmetry similar to the T4 bacteriophage tail tube and is surrounded by TssB/TssC (Ruiz et al., 2015; Park et al., 2018). It acts as a virulence factor and chaperone that can transport effectors and interact with effector substrates via its ring-shaped structure (Silverman et al., 2013). TssB, in complex with TssC, assembles cytoplasmic structures resembling a bacteriophage sheath, which can change rapidly between contracted and extended states and generate a mechanical force to drive T6SS components outside the cell (Silverman et al., 2012). VgrG proteins, which participate in the composition of the baseplate complex, are structurally similar to the puncturing device of the T4 bacteriophage (Leiman et al., 2009). Another inner membrane protein, TssM, representing homologs of the T4SS IcmF, has been shown to be required for TssD secretion; therefore, TssM is required for T6SS activity (Weber et al., 2013).

Wang et al. (Wang et al., 2018) confirmed that VgrG is involved in both antimicrobial resistance and virulence in *A. baumannii* ATCC 19606. Therefore, markers of T6SS, e.g., TssB, TssD, and TssM, were investigated in this study to determine their importance in the drug resistance and bacterial virulence of *A. baumannii*.

## 2 Materials and methods

### 2.1 *A. baumannii* clinical isolates and cultural conditions

A total of 114 A*. baumannii* clinical strains isolated from inpatient specimens were collected from the Shaanxi Provincial People’s Hospital and First Hospital of Yulin in 2018. Isolates were confirmed as *A. baumannii* by matrix-assisted laser desorption/ionization time-of-flight mass spectrometry (MALDI-TOF MS, Bruker Daltonik GmbH, Leipzig, Germany). All strains were grown on Luria-Bertani (LB, Beijing Land Bridge, Beijing, China) agar plates at 37°C for 18-24 h. When necessary, kanamycin (Aladdin, Shanghai, China) and carbenicillin (Aladdin) were added to the LB medium at final concentrations of 50 μg/mL and 100 μg/mL, respectively. The bacteria were cultured at 37°C and preserved in LB broth containing 20% glycerol at -80°C for further analysis.

### 2.2 Antimicrobial susceptibility testing

Antimicrobial susceptibility testing was performed using a VITEK 2 system (bioMérieux, Marcyl’ Étoile, France). The susceptibility results were interpreted according to the guidelines of the Clinical and Laboratory Standard Institute (CLSI) (CLSI, 2018). The resistance rate was used to reflect the resistance level of the strains to antibiotics, which is present as the percent of antibiotic resistant isolates in the total tested samples (ANRESIS, 2022).

Furthermore, the disk-diffusion method was used to detect the antibiotic sensitivity of the knockout and complementary mutants of the target genes (Wiegand et al., 2008). Fifteen categories containing thirty-five antimicrobial agents were tested, including penicillins (penicillin, PEN, 1 μg; oxacillin, OXA, 1 μg; ampicillin, AMP, 10 μg), extended-spectrum cephalosporins (ceftazidime, CAZ, 30 μg; cephalexin, LEX, 30 μg; cefazolin, CZO, 30 μg; cefradine, CED, 30 μg; cefuroxime, CXM, 30 μg; ceftriaxone, CRO, 30 μg; cefepime, FEP, 30 μg; cefotaxime, CTX, 30 μg; cefoperazone, CFP, 75 μg), carbapenems (meropenem, MEM, 10 μg; imipenem, IPM, 10 μg), aminoglycosides (gentamicin, GEN, 120 μg; tobramycin, TOB, 10 μg; amikacin, AMK, 30 μg; streptomycin, STR, 10 μg), fluoroquinolones (ciprofloxacin, CIP, 5 μg; norfloxacin, NOR, 10 μg; levofloxacin, LVX, 5 μg; ofloxacin, OFX, 5 μg), folate pathway inhibitors (trimethoprim/sulfamethoxazole, SXT, 25 μg), macrolides (erythromycin, ERY, 15 μg; midecamycin, MID, 30 μg), glycopeptide (vancomycin, VAN, 30 μg), nitrofurans (furazolidone, FRZ, 300 μg), chloramphenicols (chloramphenicol, CHL, 30 μg), lincosamides (clindamycin, CLI, 2 μg), nitroimidazoles (metronidazole, MTR), rifamycin (rifampin, RFP, 5 μg), tetracyclines (minocycline, MIN, 30 μg; tetracycline, TCY, 30 μg; doxycycline, DOX, 30 μg), and polymyxins (polymyxin B, PMB, 300 μg) (Hangzhou Microbial Reagent co., LTD, Hangzhou, China).

### 2.3 Identification of target T6SS core genes in *A. baumannii* clinical isolates

One hundred complete genomes of *A. baumannii* strains (**Supplementary Table S2**), listed in the NCBI Nucleotide database, were selected for comparative analysis of the T6SS gene cluster using Mauve. The sequences of the three T6SS core genes, *tssB*, *tssD*, and *tssM*, were aligned using SnapGene. After alignment, the phylogeny of the three core genes were constructed using the UPGMA model in MEGA, with 1000 Bootstrap replications. Two pairs of primers for each gene were designed based on the conservative clips of the selected genes. The gene targets, primer sequences, and expected amplicon sizes are shown in **Table 1**. Polymerase chain reaction (PCR) was performed to identify the three genes in 114 clinical *A. baumannii* isolates using 2 × Taq Master Mix (Novoprotein, Shanghai, China) with a total volume of 20 μL. This volume contained 10 μL of 2 × Taq Master Mix, 1 μL of each primer, and 8 μL of sterile water. The amplification program consisted of a pre-denaturation step at 94°C for 2 min, followed by 30 cycles of denaturation at 95°C for 20 s, annealing at 55°C for 20 s, and extension at 72°C for 1 min, with a final elongation at 72°C for 5 min.

**Table 1:** Primers used in this study.

Primer	Primer sequence (5’ to 3’)	Application	Product size (bp)
*tssB*	Pair 1 F: GGTGATGCGAAAGAAGCGAA R: ATTCATGATCGGCCTCCGCA Pair 2 F: TTCGACCTCCACGTGTTCAG R: GCCTCCGCACTTAATTTACGG	Detection of *tssB*	421 451
*tssD*	Pair 1 F: ATTCTGAGCACAAAGGTTGG R: CAGCTTCTTACGCAGCGTAA Pair 2 F: AAAGTTGATGGAGAATCTCG R: GCACCTTTAGCAGTACCGTT	Detection of *tssD*	466 416
*tssM*	Pair 1 F: CTGGCGTTCTTGGCTTGATG R: AGATGGCGGTTGGTTTCAGT Pair 2 F: CGCACCTACAAATGGAATGG R: TAACTCCCGCAGCAGGTTGT	Detection of *tssM*	1015 575
*kan-*FRT	F: ATTGTGTAGGCTGGAGCTGCTTC R: GGTCCATATGAATATCCTCCTTAGTTCC	Construction of Δ*tssB*::kan	1484
*tssB-*up	F: ACGCAACGCGTAATAAAGTGAGTG R: TCCAGCCTACACAATCATAAGATTC	Construction of Δ*tssB*::kan	168
*tssB-*dw	F: GGAGGATATTCATATGGACCTAATACTCAATCAGC R: GTTCATTGTGCATGATCTGAC	Construction of Δ*tssB*::kan	257
*tssB-*screen	F: TGCGTAATCAGGAAATGCGA R: GAGCGGATTTGAAGGCGTTTCC	Confirmation of Δ*tssB*::kan and Δ*tssB*	2151 667
*tssB-*comp	F: TACCAGGAGGAAACGACCGCTGTCAAA R: CAAAACAGCCAAGCTCTTCATTTCGTGC	Complementation of *tssB*	693
*tssD-*up	F: ACCCGTTACGTGAAGCGTCT R: TCCAGCCTACACAATCATTTAGAAC	Construction of Δ*tssD*::kan	204
*tssD-*dw	F: GGAGGATATTCATATGGACCTTACGCAGCG R: CCCGACTATTGAGCAAATCT	Construction of Δ*tssD*::kan	243
*tssD-*screen	F: GCAATGATGCGAGATAAAGTGGG R: GCTCTTCTATCACAACACACC	Confirmation of Δ*tssD*::kan and Δ*tssD*	2405 921
*tssD-*comp	F: TACCAGGAGGAAACGACCTCAGTCCTC R: CAAAACAGCCAAGCTGCAGAGGCTAAC	Complementation of *tssD*	620
*tssM-*up	F: GGGCATACCCAGCATTTAGTTG R: TCCAGCCTACACAATCATTCTATTCTCG	Construction of Δ*tssM*::kan	198
*tssM-*dw	F: GGAGGATATTCATATGGACCTGACATGG R: GAAGGCAACTTGCTATATGC	Construction of Δ*tssM*::kan	216
*tssM-*screen	F: GCAGCCAAAGTTCTTCATACGC R: GTAAATGACTGAGTACCATCGG	Confirmation of Δ*tssM*::kan *tssB*-screen and *tssD*-screen	2185 701
*tssM-*comp	F: TACCAGGAGGAAACGAGAAGATGATGCG R: CAAAACAGCCAAGCTCCTTGAATGTAGAGG	Complementation of *tssM*	3913

### 2.4 Construction of *tssB*, *tssD*, and *tssM* knockout mutant

For convenience of constructing the deletion mutants, the clinical strain AB795639 was selected as the wild-type strain. This strain was not only sensitive to all the tested antibiotics but also harbored T6SS genes. The *tssB*, *tssD*, or *tssM* genes were knocked out from the AB795639 genome by allelic replacement using the Rec_Ab_ system, as previously described (Tucker et al., 2014), with minor modifications. Briefly, the genomic DNA of AB795639 isolates was extracted using a TIANamp Bacteria DNA Kit (TIANGEN, Beijing, China), according to the manufacturer’s instructions. The homologous upstream and downstream fragments flanking the coding sequences of *tssB*, *tssD*, and *tssM* were amplified from AB795639, and the kanamycin resistance (Km^R^) selection marker was amplified from pKD4 by PCR using 2 × Fast Pfu Master Mix (Novoprotein). The deletion cassette of the upstream–Km^R^–downstream fragment was linked by overlapping PCR. This was subsequently electroporated into pAT02-containing competent AB795639 cells, and bacteria were grown in LB medium supplemented with 2 mM IPTG. Kan^R^-insertion mutants were selected on LB agar plates containing kanamycin and subsequently confirmed by PCR using 2 × Taq Master Mix (Novoprotein). The kanamycin cassette in successful recombinants was deleted by the pAT03 plasmid expressing the FLP recombinase. Furthermore, the target gene mutant strain was confirmed by PCR and Sanger sequencing. Deletion mutants are listed in **Table 2**.

**Table 2:** Strains used in this study.

Strain	Annotation
AB795639	AB795639 wild type strain
Δ*tssB*	AB795639 *tssB* knockout strain
Δ*tssB-*C	AB795639 *tssB* complementary strain
Δ*tssD*	AB795639 *tssD* knockout strain
Δ*tssD-*C	AB795639 *tssD* complementary strain
Δ*tssM*	AB795639 *tssM* knockout strain
Δ*tssM-*C	AB795639 *tssM* complementary strain

### 2.5 Complementation of *tssB*, *tssD*, and *tssM*

For genetic complementation of the mutant strain, the target gene was amplified from AB795639 by PCR using 2 × Fast Pfu Master Mix (Novoprotein) and the linear pAT03 plasmid was amplified from pAT03 DNA using PrimeSTAR Max DNA polymerase (TaKaRa, Beijing, China). The fragment was cloned into pAT03 using the Seamless Cloning Kit (Beyotime) and further transformed into DH5α to obtain the recombinant plasmids pAT03-*tssB*, pAT03-*tssD*, or pAT03-*tssM*. The plasmids obtained were confirmed by sequencing, followed by transformation into competent target gene-deletion mutant cells. The positive cells were selected on LB agar containing carbenicillin and IPTG. Complementation of *tssB*, *tssD*, or *tssM* was confirmed via PCR. Complementary strains are presented in **Table 2**.

### 2.6 Bacterial morphology and growth curve analysis

The morphology of AB795639 and all of the mutants was observed by Gram staining. Furthermore, the effects of *tssB*, *tssD*, and *tssM* genes on bacterial growth were determined by growth curves (Han et al., 2022). Briefly, overnight cultures of the wild-type strain, the deletion mutants, and the complemented strains were inoculated into fresh LB broth at 37°C with a concentration of 5 × 10^5^ CFU/mL. The optical density of cultured strains at 600 nm (OD_600_) was evaluated every hour from 0 to 24 h using an UNICO UV-2100 spectrophotometer (UNICO, Shanghai, China).

### 2.7 Biofilm formation assay

Biofilm production abilities of the deletion and complementary mutants, as well as their parental strains, were assayed using the crystal violet staining method as previously described (Ryu et al., 2017). Briefly, bacterial suspensions from overnight cultures were normalized to the OD_600_ of 0.2, which contained approximately 10^8^ CFU/mL bacteria. Next, 200 μL of the bacterial suspensions were seeded into 96-well plates and cultured at 37°C for 24 h. After incubation, planktonic bacteria were removed, and the plates were gently washed with phosphate buffered saline (PBS) three times and air-dried. The biofilm remaining in the well was stained with 0.1% crystal violet for 20 min. After rinsing twice with PBS, the dye attached to the biofilm was solubilized in 95% ethanol. Experiments were performed with three independent cultures, and the results were recorded as the mean ± SD of OD_570_.

### 2.8 Serum resistance assay

The serum resistance assay was slightly modified according to a previous report (Shin and Ko, 2015). Normal human serum (NHS) was collected from 10 healthy human donors. The pooled NHS were aliquoted and stored at -70°C until further use. Briefly, the overnight *A. baumannii* cultures were adjusted to an OD_600_ of 0.1 and resuspended in 1 mL of PBS. Then, 10 μL of the bacterial suspension was mixed with 100 μL normal human serum (NHS). The mixtures were incubated at 37°C for 1 h, followed by serial dilutions, which were plated on LB agar plates. Bacterial colonies were counted, and the serum bactericidal effect was expressed as the ratio of the CFUs in the serum bacterial suspension to the CFUs in a bacterial suspension without NHS. All experiments were performed in triplicate.

### 2.9 *In vitro* interbacterial competitive growth assays

Assays to determine the ability of *A. baumannii* strains to out-compete rival bacteria were performed as described previously, with minor modifications (Weber et al., 2016). Briefly, overnight cultures of different *A. baumannii* and *Escherichia coli* strains with the pWH1266 plasmid carrying a tetracycline-resistance gene were adjusted to an OD_600_ of 1.0. Then, 100 μL of *E. coli* was mixed with 10 μL of *A. baumannii*, and 10 μL of the mixture was spotted onto 0.22 μm nitrocellulose membranes overlaid on an LB agar plate. After 12 h incubation at 37°C, the spots were excised from the agar and resuspended by mixing with 1 mL of LB. The cultures underwent 10-fold serial dilutions, then, 100 μL aliquots of the selected dilutions were plated onto 16 μg/mL tetracycline-containing LB agar plates to determine the number of *E. coli* remaining. The controls consisted of *E. coli* mixed with LB medium. The assays were repeated in triplicate, and a representative image is shown.

### 2.10 *Galleria mellonella* infection assay

The *G. mellonella* infection experiment was performed as described by Harding et al. (Harding et al., 2016) with minor modifications. The different *A. baumannii* strains tested were grown in LB medium overnight and then adjusted to a final OD_600_ of 0.2. A concentration of 10^5^ CFU/mL was obtained by serial dilution of the suspensions. Groups of 20 randomly selected larvae were used in each assay. A Hamilton microliter syringe was used to inject 20 μL of the bacterial suspension into the hemolymph of each larva via the second last left proleg. As a control, one group of *G. mellonella* was injected with 20 μL sterile LB. After injection, the larvae were kept on glass plates at 37°C, and the number of dead individuals was scored approximately every 6 h until 96 h, depending on the response to physical stimuli using a sterilized tip.

### 2.11 Statistical analysis

Data analysis was performed using the GraphPad Prism software (version 8.0 (San Diego, CA, USA) and SPSS (version 22.0; SPSS Inc., Chicago, IL, USA). The relationship between the prevalence of T6SS core genes and antimicrobial resistance in *A. baumannii* clinical isolates was analyzed using the Pearson chi-square test. The survival rate of larvae was compared using the log-rank test. To determine the differences in the drug sensitivity, growth, ability to form biofilms, ability to kill rival bacterial strains, and resistance to human serum of the *A. baumannii* strains (wild type, deletion mutants, and complemented mutants), a one-way ANOVA was employed. Statistical significance was set at *P* < 0.05.

## 3 Results

### 3.1 The antimicrobial susceptibility profiles

**Figure 1:**
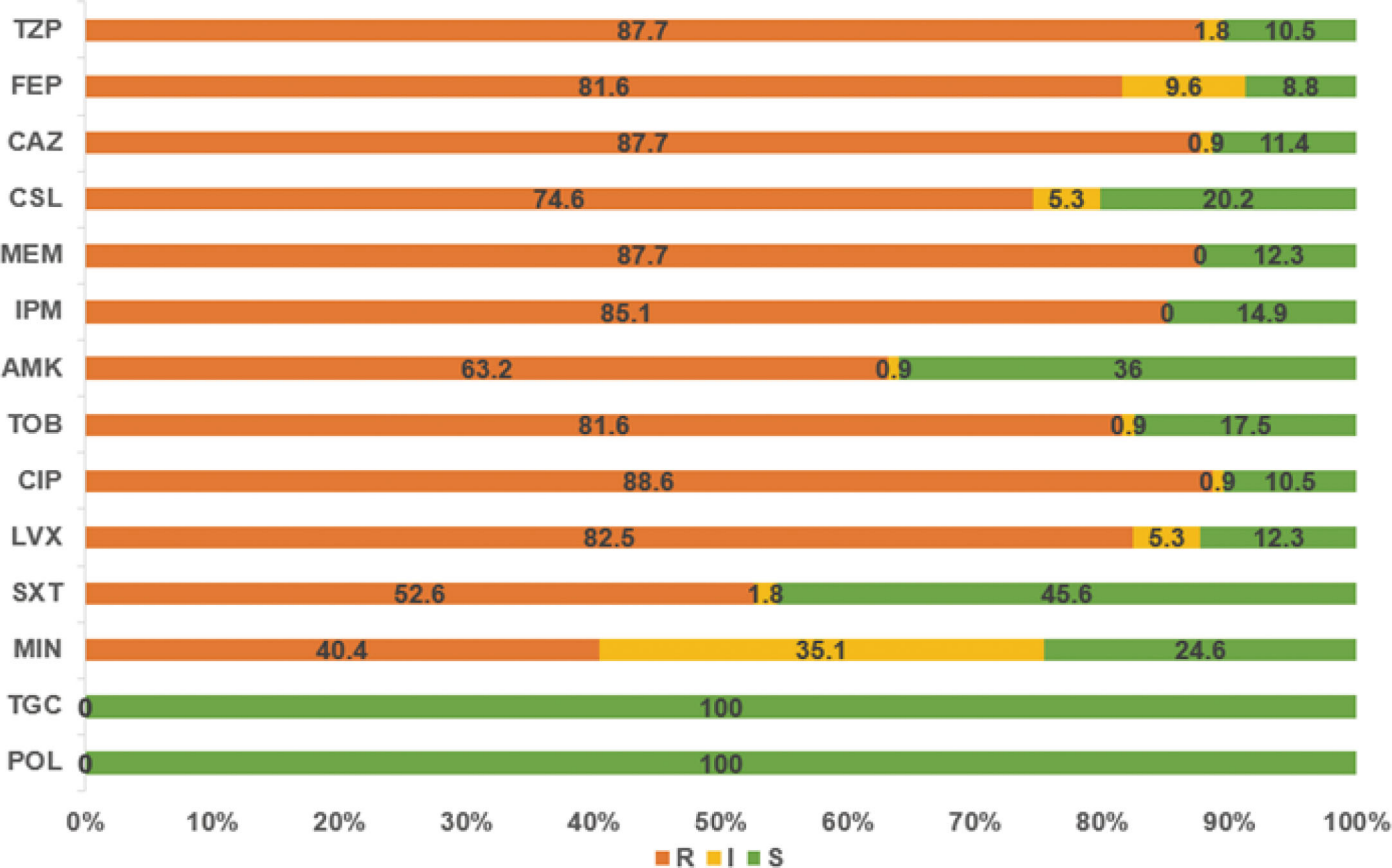
The resistance rates of *A. baumannii* strains to various antibiotics. *TZP*, piperacillin/tazobactam; *FEP*, cefepime; *CAZ*, ceftazidime; *CSL*, cefoperazone/sulbactam; *MEM*, meropenem; *IPM*, imipenem; *AMK*, amikacin; *TOB*, tobramycin; *CIP*, ciprofloxacin; *LVX*, levofloxacin; *SXT*, trimethoprim/sulfamethoxazole; *MIN*, minocycline; *TGC*, tigecycline; *POL*, polymyxin.

**Figure 2:**
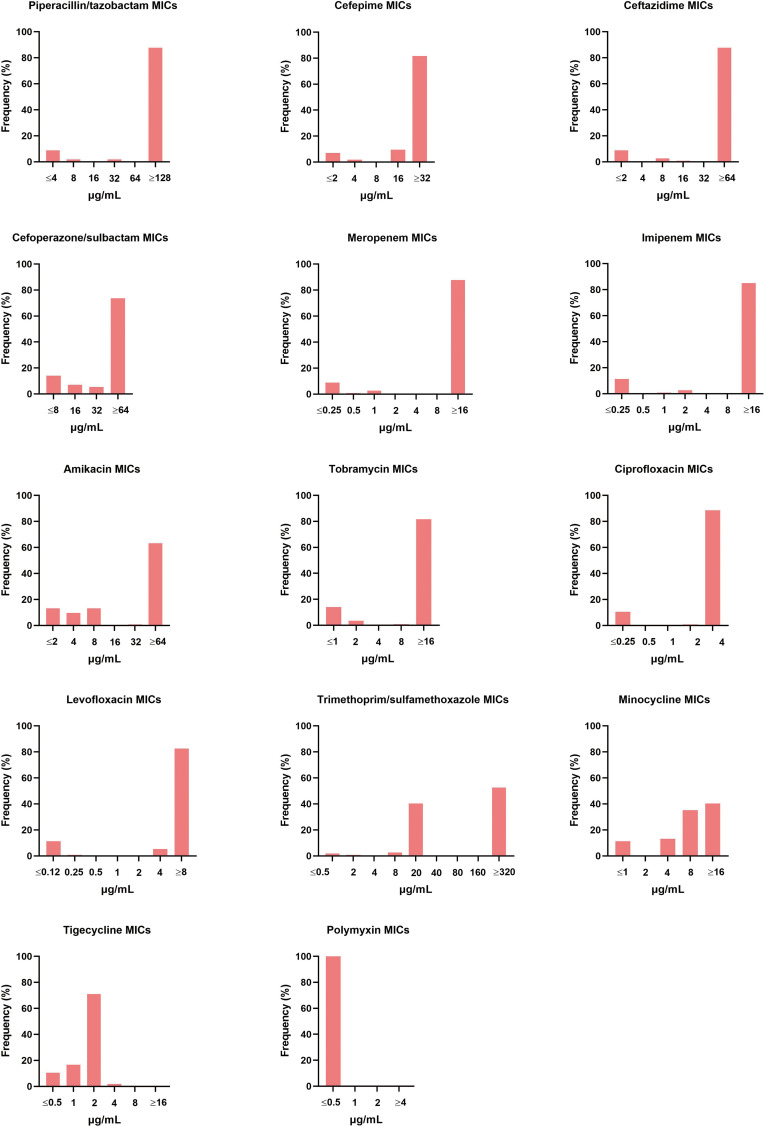
Distribution of different antibiotic MICs. The MIC breakpoints for each of the antibiotics tested was as per the guidelines of the Clinical and Laboratory Standard Institute.

The 114 A*. baumannii* clinical isolates were resistant to multiple antibiotics (**Supplementary Table S1**). The strains possessed the highest resistance rate against ciprofloxacin (88.6%). This was followed by meropenem, ceftazidime, piperacillin/tazobactam, imipenem, levofloxacin, cefepime, and tobramycin, with resistance rates of over 80%. The resistance rates of minocycline, trimethoprim/sulfamethoxazole, amikacin, and cefoperazone/sulbactam were slightly lower, that from 40.4% to 74.6%. Only tigecycline and polymyxin showed high activity against *A. baumannii* (**Figure 1**). Among the isolates, six were susceptible to all of the tested antimicrobial agents. Eleven strains were resistant to all antimicrobial agents except tigecycline and polymyxin. The distribution of minimum inhibitory concentrations (MICs) of the antibiotics tested in this study is shown in **Figure 2**.

### 3.2 Conservation of *tssB*, *tssD* (*hcp*), and *tssM* in *A. baumannii* genomes and their prevalence in *A. baumannii* clinical isolates.

We first selected 100 complete genome sequences of *A. baumannii* containing T6SS from GenBank and analyzed the T6SS locus. Comparative analysis using Mauve indicated that the T6SS gene cluster is effectively conserved among various *A. baumannii* isolates (**Supplementary Figure S1**). The positions of the three core genes, *tssB*, *tssD*, and *tssM*, in each genome are listed in **Supplementary Table S2**. Thereafter, the *tssB*, *tssD*, and *tssM* from the 100 genomes were aligned. According to the analysis using MEGA (**Supplementary Figure S2**) and SnapGene (**Supplementary Figures S3**–**S5**), the sequences of *tssB*, *tssD*, and *tssM* genes were highly conserved among these 100 A*. baumannii* strains.

The highly conservative nature of the core genes makes it possible to analyze their prevalence in clinical isolates. As is shown in **Supplementary Table S1**, 77.2% (88/114) of the clinical strains harbored all three genes. Moreover, 2.63% (3/114) and 8.77% (10/114) of the isolates were positive for two genes and one gene, respectively. For the remaining 11.4% (13/114) of isolates, the absence of T6SS was confirmed using both pairs of primers for each gene.

### 3.3 The relationship between T6SS core genes and antibiotic resistance in *A. baumannii* clinical isolates

As T6SS has been found to be closely related to antibiotic resistance (Li et al., 2023), the correlation between each core gene and the antibiotic resistance data for the clinical strains was analyzed (**Table 3**). It was found that *tssB* correlated with resistance to all antibiotics (*P* < 0.05), except for tigecycline and polymyxin. Specifically, the resistance rate of strains with *tssB* was higher than that without *tssB*. Similar results were observed for *tssD*. In addition, *tssM* also showed correlation in terms of antibiotic resistance with all drugs except meropenem, amikacin, tigecycline, and polymyxin.

**Table 3:** The correlation analysis between individual core gene and antimicrobial resistance.

Resistant antibiotics	*tssB* (+)	*tssB* (-)	*P*	*tssD* (+)	*tssD* (-)	*P*	*tssM* (+)	*tssM* (-)	*P*
	n=92	n=22		n=91	n=23		n=97	n=17	
TZP	89 (96.7)	11 (50.0)	0.000***	89 (97.8)	11 (47.8)	0.000***	89 (91.8)	11(64.7)	0.004**
FEP	84 (91.3)	9 (40.9)	0.000***	83 (91.2)	10 (43.5)	0.000***	84 (86.6)	9 (52.9)	0.003**
CAZ	89 (96.7)	11 (50.0)	0.000***	89 (97.8)	11 (47.8)	0.000***	89 (91.8)	11(64.7)	0.007**
CSL	78 (84.8)	7 (31.8)	0.000***	79 (86.8)	6 (26.1)	0.000***	78 (80.4)	7 (41.2)	0.001**
MEM	88 (95.7)	12 (54.5)	0.000***	88 (96.7)	12 (52.2)	0.000***	88 (90.7)	12 (70.6)	0.053
IPM	87 (94.6)	10 (45.5)	0.000***	87 (95.6)	10 (43.5)	0.000***	86 (88.7)	11 (64.7)	0.021*
AMK	65 (70.7)	7 (31.8)	0.001**	65 (71.4)	7 (30.4)	0.001**	64 (66.0)	8 (47.1)	0.294
TOB	84 (89.1)	9 (40.9)	0.000***	84 (92.3)	9 (39.1)	0.000***	83 (85.6)	10 (58.8)	0.021*
CIP	90 (97.8)	11 (50.0)	0.000***	90 (98.9)	11 (47.8)	0.000***	91 (93.8)	10 (58.8)	0.000***
LVX	85 (92.4)	9 (40.9)	0.000***	85 (93.4)	9 (39.1)	0.000***	87 (89.7)	7 (41.2)	0.000***
SXT	55 (59.8)	5 (22.7)	0.003**	54 (59.3)	6 (26.1)	0.006**	55 (56.7)	5 (29.4)	0.048*
MIN	40 (43.5)	6 (27.3)	0.000***	41 (45.1)	5 (21.7)	0.000***	42 (43.3)	4 (23.5)	0.013*
TGC	0	0	—	0	0	—	0	0	—
POL	0	0	—	0	0	—	0	0	—

Categorical variables are expressed as n (%) and were analyzed using the chi-square test (Fisher probabilistic method). TZP, piperacillin/tazobactam; FEP, cefepime; CAZ, ceftazidime; CSL, cefoperazone/sulbactam; MEM, meropenem; IPM, imipenem; AMK, amikacin; TOB, tobramycin; CIP, ciprofloxacin; LVX, levofloxacin; SXT, trimethoprim/sulfamethoxazole; MIN, minocycline; TGC, tigecycline; POL, polymyxin. *, P < 0.05; **, P < 0.01; ***, P < 0.001.

Further analysis was performed to explore the relationship between the core gene combinations and antimicrobial resistance (**Table 4**). Interestingly, every type of combination showed strong correlation with all antibiotics (*P* < 0.001), except for amikacin and trimethoprim/sulfamethoxazole (*P* < 0.05). Owing to the full susceptibility of all *A. baumannii* clinical isolates to tigecycline and polymyxin, there was no correlation between the three core genes and these two antibiotics.

**Table 4:** The correlation analysis between core gene combinations and antimicrobial resistance.

Resistant antibiotics	*tssB*+*D*		*P*	*tssB*+*M*		*P*	*tssD*+*M*		*P*	*tssB*+*D*+*M*		*P*
	(+) n=90	(-) n=24		(+) n=89	(-) n=25		(+) n=88	(-) n=26		(+) n=88	(-) n=26	
TZP	88 (97.8)	12 (50.0)	0.000***	86 (96.6)	14 (56.0)	0.000***	86 (97.7)	14 (53.8)	0.000***	86 (97.7)	14 (53.8)	0.000***
FEP	83 (92.2)	10 (41.7)	0.000***	81 (91.0)	12 (48.0)	0.000***	81 (92.0)	12 (46.2)	0.000***	81 (92.0)	12 (46.2)	0.000***
CAZ	88 (97.8)	12 (50.0)	0.000***	86 (96.6)	14 (56.0)	0.000***	86 (97.7)	14 (53.8)	0.000***	86 (97.7)	14 (53.8)	0.000***
CSL	78 (86.7)	7 (29.2)	0.000***	76 (85.4)	9 (36.0)	0.000***	76 (86.4)	9 (34.6)	0.000***	76 (86.4)	9 (34.6)	0.000***
MEM	87 (96.7)	13 (54.2)	0.000***	85 (95.5)	15 (60.0)	0.000***	85 (96.6)	15 (57.7)	0.000***	85 (96.6)	15 (57.7)	0.000***
IPM	86 (95.6)	11 (45.8)	0.000***	84 (94.4)	13 (52.0)	0.000***	84 (95.5)	13 (50.0)	0.000***	84 (95.5)	13 (50.0)	0.000***
AMK	64 (71.1)	8 (33.3)	0.001**	62 (69.7)	10 (40.0)	0.014*	62 (70.5)	10 (38.5)	0.006**	62 (70.5)	10 (38.5)	0.006**
TOB	83 (92.2)	10 (41.7)	0.000***	81 (91.0)	12 (48.0)	0.000***	81 (92.0)	12 (46.2)	0.000***	81 (92.0)	12 (46.2)	0.000***
CIP	89 (98.9)	12 (50.0)	0.000***	87 (97.8)	14 (56.0)	0.000***	87 (98.9)	14 (53.8)	0.000***	87 (98.9)	14 (53.8)	0.000***
LVX	84 (93.3)	10 (41.7)	0.000***	83 (93.3)	11 (44.0)	0.000***	83 (94.3)	11 (42.3)	0.000***	83 (94.3)	11 (42.3)	0.000***
SXT	54 (60.0)	6 (25.0)	0.004**	54 (60.7)	6 (24.0)	0.002**	52 (59.1)	8 (30.8)	0.021*	53 (60.2)	7 (26.9)	0.021*
MIN	40 (44.4)	6 (25.0)	0.000***	40 (44.9)	6 (24.0)	0.000***	41 (46.6)	5 (19.2)	0.000***	40(45.5)	6 (23.1)	0.000***
TGC	0	0	—	0	0	—	0	0	—	0	0	—
POL	0	0	—	0	0	—	0	0	—	0	0	—

Categorical variables are expressed as n (%) and were analyzed using the chi-square test (Fisher probabilistic method). TZP, piperacillin/tazobactam; FEP, cefepime; CAZ, ceftazidime; CSL, cefoperazone/sulbactam; MEM, meropenem; IPM, imipenem; AMK, amikacin; TOB, tobramycin; CIP, ciprofloxacin; LVX, levofloxacin; SXT, trimethoprim/sulfamethoxazole; MIN, minocycline; TGC, tigecycline; POL, polymyxin. *, P < 0.05; **, P < 0.01; ***, P < 0.001.

### 3.4 Effects of *tssB*, *tssD*, and *tssM* on antibiotic resistance of *A. baumannii*

**Figure 3:**
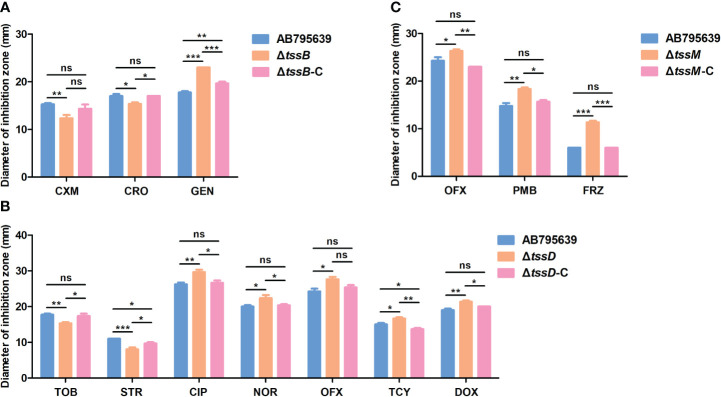
Antibiotic susceptibilities of AB795639 using the deletion and complementary mutants of three T6SS core genes as assessed by disk diffusion. The changed antibiotic susceptibility related to *tssB* **(A)**, *tssD* **(B)**, and *tssM* **(C)**. *CXM*, cefuroxime; *CRO*, ceftriaxone; *GEN*, gentamicin; *TOB*, tobramycin; *STR*, streptomycin; *CIP*, ciprofloxacin; *NOR*, norfloxacin; *OFX*, ofloxacin; *TCY*, tetracycline; *DOX*, doxycycline; *PMB*, polymyxin B; FRZ, furazolidone. The experiment was repeated at least in triplicate, and the results are shown as means ± SD. ns, *P* > 0.05; *, *P* < 0.05; **, *P* < 0.01; ***, *P* < 0.001.

To better understand the antimicrobial roles that *tssB*, *tssD*, and *tssM* play, we constructed deletion mutants and complementary strains from the sensitive strain AB795639, which harbored all three core genes (**Table 2**). The sensitivity of the mutants to 35 antibiotics was determined to comprehensively determine the contribution of the three T6SS core genes to drug resistance in *A. baumannii*. The disk diffusion method was performed instead of MIC detection to better observe slight changes in drug resistance. Compared with the wild-type strain, the inhibition zone of Δ*tssB* by cefuroxime and ceftriaxone was reduced (*P* = 0.009 and *P* = 0.010, respectively); however, that of gentamicin was remarkably increased (*P* = 0.000) (**Figure 3A**).

As is shown in **Figure 3B**, *tssD* was the most relevant gene for antimicrobial susceptibility as the inhibition zone of the seven antibiotics changed. This demonstrated that the strain became more resistant to tobramycin (*P* = 0.004) and streptomycin (*P* = 0.000), but more sensitive to ciprofloxacin (*P* = 0.004), norfloxacin (*P* = 0.020), ofloxacin (*P* = 0.011), tetracycline (*P* = 0.016), and doxycycline (*P* = 0.002) when *tssD* was missing.

Meanwhile, the mutant strain Δ*tssM* displayed an increased susceptibility to ofloxacin (*P* = 0.033), polymyxin B (*P* = 0.002), and furazolidone (*P* = 0.000) (**Figure 3C**).

When the *tssB*, *tssD*, and *tssM* genes were complemented (**Figure 3**), the antimicrobial sensitivity of the complementary strains was recovered or partly restored to the level of the wild-type strain. The antimicrobial susceptibility results for all strains are shown in **Supplementary Table S3**.

### 3.5 The effects of three T6SS core genes on the bacterial morphology, growth, and biofilm formation in *A. baumannii*

**Figure 4:**
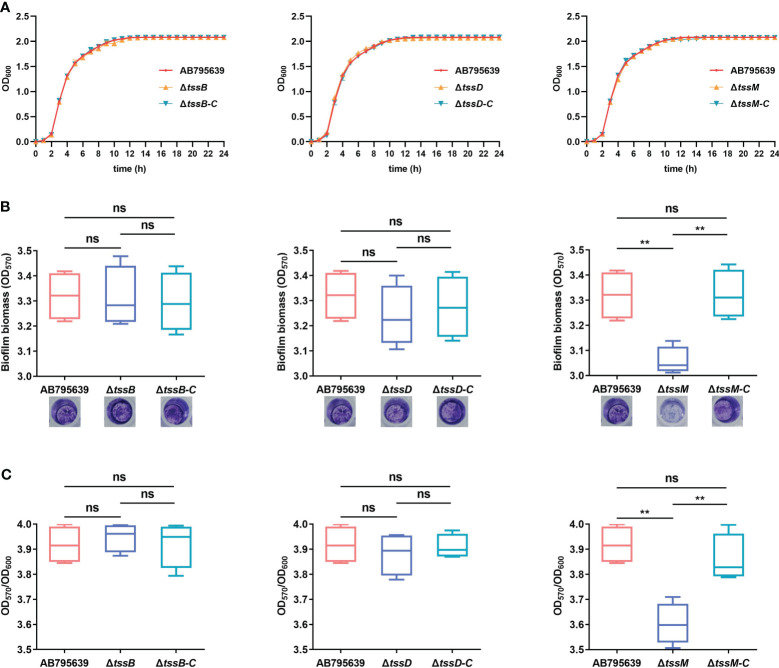
Effects of *tssB*, *tssD*, and *tssM* on bacterial growth and biofilm formation in *A baumannii*. **(A)** The growth curves of the wild-type strain, deletion mutants, and the respective complemented strains in LB broth. OD_600_ values of the bacterial cultures grown at 37°C with shaking at 200 rpm were recorded during 0-24 (h) The experiment was repeated in triplicate, and the results were presented as means ± SD. **(B)** Biofilm formation ability of AB795639, and the deletion and complementary mutants of three T6SS core genes. Biofilm biomass measured by crystal violet staining using the optical density at 570 nm (OD_570_) after 24 h incubation in strains expressing *tssB*, *tssD*, and *tssM*, in addition to a strain not expressing these genes. **(C)** The ratio of OD_570_/OD_600_ was evaluated for all the tested strains to avoid the bias caused by dissimilar bacterial numbers. The experiment was repeated in triplicate, and the results are shown as means ± SD. ns, *P* > 0.05; *, *P* < 0.05; **, *P* < 0.01.

Loss of *tssB*, *tssD*, or *tssM* did not affect either the morphology (**Supplementary Figure S6**) or the growth of AB795639 in LB broth (**Figure 4A**). The growth curves of three deletion mutants were similar to those of the wild type and the complementary strains.

In *A. baumannii*, biofilm formation is an important factor that involves in antibiotic resistance and bacterial virulence. Hence, the role of TssB, TssD, and TssM in biofilm formation by *A. baumannii* was investigated. Following 24 h of incubation, only the Δ*tssM* mutant exhibited a decreased ability to adhere to the well surfaces, suggesting a lower degree of biofilm production (*P* = 0.002). Complementation of *tssM* restored the biofilm-forming ability. In contrast, for *tssB* and *tssD*, no significant difference was observed for strains expressing these genes (*P* > 0.05) (**Figures 4B, C**). These results indicate that *tssM* is required for biofilm formation of this strain.

### 3.6 T6SS core genes *tssD* and *tssM* are required by *A. baumannii* for human serum resistance

**Figure 5:**
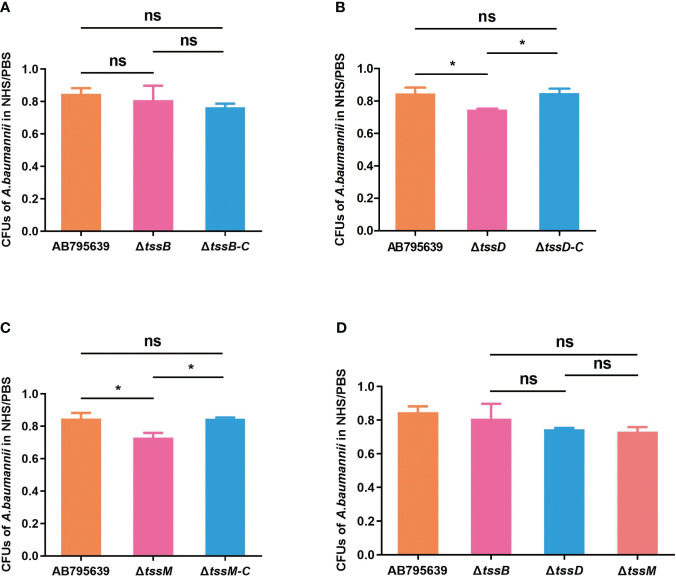
The human serum resistance ability of AB795639, the deletion and complementary mutants of three T6SS core genes. The viability ratios of strains for *tssB* **(A)**, *tssD* **(B)** and *tssM* **(C)** were calculated (colony forming units (CFUs) of bacteria in serum/CFUs of bacteria in phosphate buffer saline (PBS)). **(D)** Comparison of viability ratios among deletion mutants of three core genes. The experiment was repeated in triplicate, and the results are shown as means ± SD. ns, *P* > 0.05; *, *P* < 0.05.

By avoiding the bactericidal activity of serum, pathogenic bacteria can invade and survive in certain hosts, thus causing disease (Kim et al., 2009). Therefore, the effects of the three T6SS core genes on serum resistance were determined (**Figure 5**). The survival of *A. baumannii* in the presence of NHS differed significantly between the wild-type and mutant strains, except for Δ*tssB* (*P* > 0.05). Specifically, Δ*tssD* and Δ*tssM* mutant strains showed remarkably decreased survival after 1 h incubation with normal human serum compared with the wild-type strain (*P* = 0.033 and *P* = 0.020, respectively). The complementation of *tssD* and *tssM* restored this ability.

### 3.7 *A. baumannii* strain AB795639 outcompetes *Escherichia coli* that is more highly dependent on TssD and TssM

**Figure 6:**
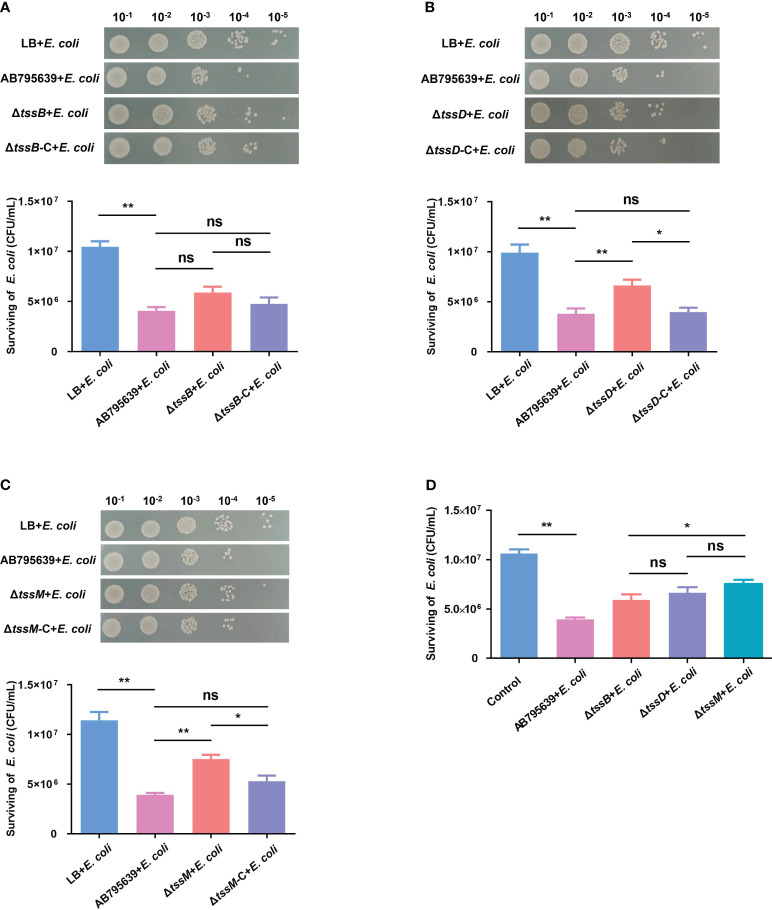
Effects of three T6SS core genes of *A baumannii* AB795639 in terms of outcompeting *E coli*. Representative images show the survival of *E coli* after incubation in LB medium (control) or with *A baumannii* strains: wild type, Δ*tssB* **(A)**, Δ*tssD* **(B)**, and Δ*tssM* **(C)**, as well as respective complemented strains. **(D)** Comparison of *E coli* survival after competition with different mutants. The experiment was repeated in triplicate, and the results are shown as means ± SD. ns, *P* > 0.05; *, *P* < 0.05; **, *P* < 0.01.

T6SS has been shown to mediate interbacterial antagonism and confer a competitive advantage in different bacteria (Basler et al., 2013; Russell et al., 2014; Zhao et al., 2018; Hsieh et al., 2019); however, less is known about the role of the three core genes in terms of these effects, especially in *A. baumannii*. The competition assays showed that when co-incubated with AB795639, the *E. coli* bacterial counts were remarkably reduced compared with those incubated in LB medium. This indicates a strong capability of this wild-type strain to outcompete other bacterial strains (**Figure 6**). The lack of *tssB* did not significantly reduce the competitive ability of AB795639 cells (**Figure 6A**). However, Δ*tssD* and Δ*tssM* weakened this ability, in contrast to AB795639 (*P* = 0.008 and *P* = 0.001, respectively). Complementation of *tssD* and *tssM* restored the ability of this bacterial to kill *E. coli*. This ability was at a similar level to the wild-type strain (**Figures 6B, C**). Among these core genes, strains expressing *tssM* showed the strongest competitive ability, followed by *tssD* (**Figure 6D**). In conclusion, killing of *E. coli* by AB795639 is dependent on a functional T6SS, and TssD and TssM play a predominant role in this process.

### 3.8 The Δ*tssD* and Δ*tssM* mutants have attenuated virulence in a *G. mellonella* infection model

**Figure 7:**
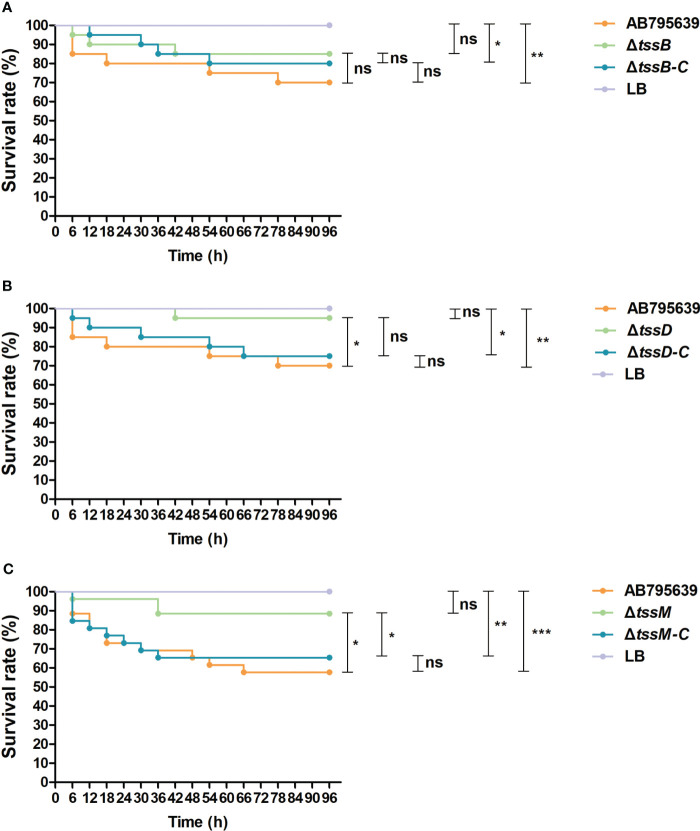
Survival of *Galleria mellonella* larvae infected with AB795639, the deletion and complementary mutants of three T6SS core genes. Kaplan–Meier survival curves of *G mellonella* infected with 1.0 × 10^5^ CFU of different *A baumannii* strains expressing *tssB* **(A)**, *tssD* **(B)** and *tssM* **(C)**, and without these genes, are shown. ns, *P* > 0.05; *, *P* < 0.05; **, *P* < 0.01; ***, *P* < 0.001.

To assess the role of *tssB*, *tssD*, and *tssM* in the virulence of *A. baumannii*, *G. mellonella* was infected with variants of these core genes as well as the wild-type strain. Larval viability was monitored every 6 h, and death was recorded. By the end of the 4-day study, the survival rate of larvae infected by Δ*tssD* and Δ*tssM* strains was significantly higher than that of the wild type strain AB795639, demonstrating remarkably attenuated virulence (*P* = 0.039 and *P* = 0.012, respectively). When the worms were inoculated with the corresponding complementary strains, larval survival was reduced or partly reduced to the level of the wild-type strain, indicating the restoration of virulence. However, no obvious difference in *G. mellonella* viability was observed for Δ*tssB* with respect to gene expression in the isolates. The control group of worms injected with sterile LB medium displayed 100% survival throughout the course of the study. Based on these data, we found that T6SS-dependent virulence of *A. baumannii* AB795639 exhibited a greater reliance on TssD and TssM (**Figure 7**).

## 4 Discussion

The challenge in treating *A. baumannii* infections is primarily attributed to the ability of *A. baumannii* to develop resistance to the last line of antibacterial agents, including carbapenems. As expected, 114 clinical isolates of *A. baumannii* showed high rates of resistance to various antimicrobials. The resistance rate of *A. baumannii* to carbapenems is still high. Most studies on the mechanisms employed by *A. baumannii* to combat antibiotics have focused on permeability defects, efflux pumps, resistance genes harbored on genetic islands, and target mutations (Elhosseiny and Attia, 2018). Secondary to these well-known mechanisms, the important virulence factor T6SS had once been reported to be related to antibiotic resistance (Liao et al., 2022). In this study, a close relationship between the presence of T6SS and antimicrobial resistance in our clinical strains was found, with a higher presence of T6SS in drug-resistant *A. baumannii* strains than in sensitive isolates. This was consistent with the latest report by Dong et al. (Dong et al., 2022).

Few studies have investigated the role of T6SS core genes in drug resistance. Wang et al. reported that a lack of *vgrG* in *A. baumannii* increased antimicrobial resistance to ampicillin/sulbactam, but reduced resistance to chloramphenicol (Wang et al., 2018). We further determined the roles of other core genes in drug resistance by constructing deletion mutants and complementary strains from a representative strain. Initially, there was a concern that the resistance mechanisms employed by drug-resistant *A. baumannii* mainly work in a high activity that may offset the effect of missing a single T6SS core gene. Indeed, according to our previous detection (Zhang et al., 2023), the majority of our tested strains had hydrolysis enzymes including carbapenemases, which remarkably increased the MIC values. In addition, since the drug adaption will provide more opportunity for bacteria to survive and further become resistant strains, T6SS may contribute to this process according to our observation that there was a correlation between drug resistance and T6SS existence. In these cases, the sensitive strain AB795639 was chosen rather than a resistant strain to conduct the following experiments for observing positive results. To comprehensively understand the role of different T6SS core genes contributing to drug resistance in *A. baumannii*, many more antibiotics (35 antibiotics) than those recommended by the CLSI (CLSI, 2018) were tested. Generally speaking, the sensitivity of AB795639 to 12 antibiotics was, to some extent, affected by the loss of individual T6SS core genes. Among these antimicrobials, cefuroxime and ceftriaxone belong to the cephalosporins class of antibiotics; gentamicin, tobramycin, and streptomycin are aminoglycosides antibiotics; ciprofloxacin, norfloxacin, and ofloxacin are fluoroquinolones; tetracycline and doxycycline are tetracyclines; polymyxin B is a member of the polymyxin family; and furazolidone belongs to the nitrofuran family. Strains lacking the T6SS core genes became more sensitive to the majority of these antibiotics, with the exception of resistance to cefuroxime, ceftriaxone, tobramycin, and streptomycin, Specifically, although extreme changes in sensitivity and resistance were not observed in the three mutants, obvious differences among inhibition zones were recorded. The Δ*tssD* strain showed the most significant changes; it was more resistant to aminoglycosides and more sensitive to fluoroquinolones and tetracyclines. This may be due to its unique inner-tube-like structure, which assembles onto the base of VgrG and extends into the cytoplasm (Li et al., 2023), and this may facilitate interactions with more antibiotics. The strain without *tssB* became more resistant to cephalosporins. However, in contrast to Δ*tssD*, Δ*tssB* became more sensitive to aminoglycosides. This difference may be related with the tail tube/sheath complex structure of Hcp/TssB. For Δ*tssM*, the mutant strain demonstrated an increased susceptibility to fluoroquinolones, polymyxins and nitrofurans. This might be because TssM is involved in Hcp recruitment and secretion. Based on these results, the syringe-shaped apparatus of T6SS may also be primarily employed by *A. baumannii* to adapt to environmental antibiotic stress.

Biofilm formation is another crucial ability that contributes to both antibiotic resistance and bacterial persistence in healthcare settings (Di Domenico et al., 2017). Apart from the commonly reported factors such as porins, capsular polysaccharides, quorum sensing, etc. (Gaddy et al., 2009; Harding et al., 2018), the association of secretion systems such as T1SS (Bap) and T5SS (Ata) with biofilm formation has also been identified (Harding et al., 2017; Elhosseiny and Attia, 2018). A few T6SS components have been reported to be associated with the biofilm formation of other pathogens (Aschtgen et al., 2008; Zhang et al., 2014). However, the role of T6SS in the biofilm formation of *A. baumannii* remains unclear. As reported by Kim et al., *A. baumannii* T6SS+ isolates formed a significantly greater biofilm mass than the T6SS− isolates (Kim et al., 2017); however, the opposite result was observed by Dong et al. (Dong et al., 2022). The biofilm forming ability of *A. baumannii* may be strain specific. Although a remarkable increased biofilm was obtained when *hcp* was absent from T6SS+ *A. baumannii* A152 (Dong et al., 2022), our results showed that *hcp* was not involved in biofilm formation in the AB795639 strain. Similarly, no difference in biofilm quantity was observed between *tssM-*lacking and harboring strains in either 17978 or DSM30011 (Weber et al., 2013; Repizo et al., 2015). However, in our study, a significant reduction in biofilm biomass was observed in the Δ*tssM* mutant compared to that in the *tssM*-expressing strains. TssM, which is involved in Hcp recruitment and secretion, is important for the activity of T6SS (Li et al., 2023). Thus, it is rational to assume that this wound exhibits a stronger biofilm-forming activity than the other two components. The contribution of the *tssB* gene to biofilm formation in *A. baumannii* has not yet been reported. In this study, we demonstrated that *tssB* did not affect biofilm formation in AB795639.

Although T6SS is a well-known multifunctional virulence apparatus in *A. baumannii*, so far, the most comprehensively studied core proteins are Hcp and VgrG. Little is known about the roles of other core proteins in terms of bacterial virulence.

Resistance to human serum is related to the mortality of patients infected with *A. baumannii*. This mechanism makes this bacterium a successful pathogen (Liao et al., 2007). Improved survival of *A. baumannii* clinical isolates harboring T6SS in the presence of NHS has been demonstrated (Repizo et al., 2017; Kim et al., 2017). However, only Hcp has been shown to play a role in serum resistance (Dong et al., 2022). In this study, we not only confirmed the importance of Hcp contributing to NHS resistance but also confirmed the role of TssM in this phenomenon. In contrast, TssB does not display this function in *A. baumannii*.

In order to colonize medical device surfaces, thrive, and eventually cause diseases in patients, a mixed population of bacteria would compete against co-existing bacteria to maintain the species. As a “molecular syringe”, T6SS is an efficient weapon with regard to the competition among bacterial species through the contact-dependent injection of effectors, interfering with vital cellular processes (Hachani et al., 2016). For *A. baumannii*, T6SS is involved in the competition with not only *E. coli*, but also with some nosocomial pathogens, e.g., *P. aeruginosa* and *K. pneumoniae* (Repizo; Repizo et al., 2015; Kim et al., 2017). This function relies on the core genes *tssD* and *tssM*; AB795639 lacking these two genes showed reduced ability to outcompete *E. coli*. This finding is consistent with that of previous studies. Carruthers et al. (Carruthers et al., 2013) demonstrated that the ability of *A. nosocomialis* M2 to kill *E. coli* is dependent on *hcp*. Furthermore, the Hcp-secreting variant of a clinical *A. baumannii* strain Ab_04_ can kill *E. coli* and *K. pneumoniae*; whereas, the non-Hcp-secreting variant from the same strain loses this ability (Weber et al., 2015). The importance of Hcp in competitive virulence might be dependent on its structure, namely, the inner tube of T6SS (Li et al., 2023). In addition, as a key component involving in the recruitment and secretion of Hcp (Ma et al., 2012), the positive effect of TssM on bacterial competition is expected. Similar to our results, *tssM*-dependent competition against *E. coli* was observed in various *A. baumannii* strains (Repizo et al., 2015; Fitzsimons et al., 2018). Interestingly, due to strain specificity, *tssM* was not implicated in terms of competition in the ATCC17978 strain (Weber et al., 2013). For TssB, losing its encoding gene resulted in an attenuated trend toward competition, while the difference was not significant. This may be due to the special structure of TssB, which encloses the Hcp tube that has less of an effect on direct virulence.

To verify the virulence of the three T6SS core genes beyond bacterial killing, we infected *G. mellonella* with various mutants. *G. mellonella* is the caterpillar of the greater wax moth and has been widely used to study the virulence mechanisms of several human pathogens, including *A. baumannii* (Jander et al., 2000; Peleg et al., 2009; Harding et al., 2012; Iwashkiw et al., 2012; Heindorf et al., 2014; Jacobs et al., 2014; Tsai et al., 2016). The innate immune system of *G. mellonella* shares a high degree of homology with that of other mammals. Consistent with our *in vitro* results, TssD and TssM contributed to the virulence of *A. baumannii*, and the loss of these two components dramatically increased the survival of the infected *G. mellonella*. Nevertheless, no significant difference was observed between the *tssB*-deletion mutant and the expressing strains. This demonstrated that TssB, which forms a helical sheath with TssC around the Hcp tube, plays a less important role in host colonization.

Consequently, we determined the effects of *tssB*, *tssD*, and *tssM* in terms of drug resistance and virulence in the sensitive clinical strain AB795639. However, additional studies are needed to confirm the roles of the three T6SS core genes in *A. baumannii* due to strain specificity. In addition, further research will be required to explore the roles of other T6SS core genes, as well as to determine the mechanisms of action and regulation of *tssB*, *tssD*, and *tssM.*

## 5 Conclusion

The prevalence of T6SS is high among clinical *A. baumannii* isolates. This study explored the finding indicating there was a high correlation between T6SS carriage and multidrug resistance in *A. baumannii*. T6SS may help bacteria to demonstrate increased drug resistance by enhancing horizontal gene transfer among bacteria (Dong et al., 2022). More interestingly, the existence of T6SS could contribute to intrinsic resistance to specific drugs such as gentamicin, ciprofloxacin, norfloxacin, ofloxacin, tetracycline, doxycycline, polymyxin B, and furazolidone.

Furthermore, we evaluated the roles of three T6SS core components, TssB, TssD (Hcp), and TssM, in terms of biofilm formation, bacterial competition, normal human serum resistance, and host colonization. TssM was the most important factor that participated in all of these virulence phenomena. This was followed by TssD, which was negative for biofilm formation. TssB has less of an effect on either biofilm formation or bacterial virulence.

This information may provide potential therapeutic and vaccine targets to control *A. baumannii* infections. Further studies are warranted to explore the roles of other T6SS core genes.

## Data availability statement

The original contributions presented in the study are included in the article/**Supplementary Materials**, further inquiries can be directed to the corresponding author/s.

## Author contributions

PL: Conceptualization, Data curation, Formal analysis, Investigation, Methodology, Project administration, Software, Validation, Visualization, Writing – original draft, Writing – review & editing. SZ: Methodology, Writing – review & editing. JW: Methodology, Writing – review & editing. MMA: Methodology, Writing – review & editing. KL: Methodology, Writing – review & editing. SL: Methodology, Writing – review & editing. PM: Resources, Writing – review & editing. XW: Resources, Writing – review & editing. HL: Methodology, Writing – review & editing. HT: Methodology, Writing – review & editing. BH: Project administration, Supervision, Writing – review & editing. JL: Funding acquisition, Writing – review & editing. SH: Funding acquisition, Writing – review & editing. LH: Conceptualization, Data curation, Funding acquisition, Investigation, Methodology, Project administration, Resources, Supervision, Validation, Visualization, Writing – original draft, Writing – review & editing.

## Supplementary material

Supplementary Figure 1 Comparative analysis of the T6SS gene cluster from the *A. baumannii* genomes collected from Genbank.

Supplementary Figure 2 Phylogeny of three T6SS core genes *tssB* **(A)**, *tssD* **(B)** and *tssM* **(C)** from the *A. baumannii* genomes collected from Genbank were constructed by UPGMA model in MEGA, with the Bootstrap replications as 1000.

Supplementary Figure 3 The sequence alignment of *tssB* gene from the *A. baumannii* genomes collected from Genbank.

Supplementary Figure 4 The sequence alignment of *tssD* gene from the *A. baumannii* genomes collected from Genbank.

Supplementary Figure 5 The sequence alignment of *tssM* gene from the *A. baumannii* genomes collected from Genbank.

Supplementary Figure 6 Representative Gram-stain results of AB795639, the deletion and complementary mutants of three T6SS core genes. Scale bars: 100 pixels.
