# Supplementary figures and images for: The role of type VI secretion system genes in antibiotic resistance and virulence in Acinetobacter baumannii clinical isolates
Source: Front Cell Infect Microbiol. 2024 Feb 7;14:1297818. doi: 10.3389/fcimb.2024.1297818 (PMC10879597; doi:10.3389/fcimb.2024.1297818)

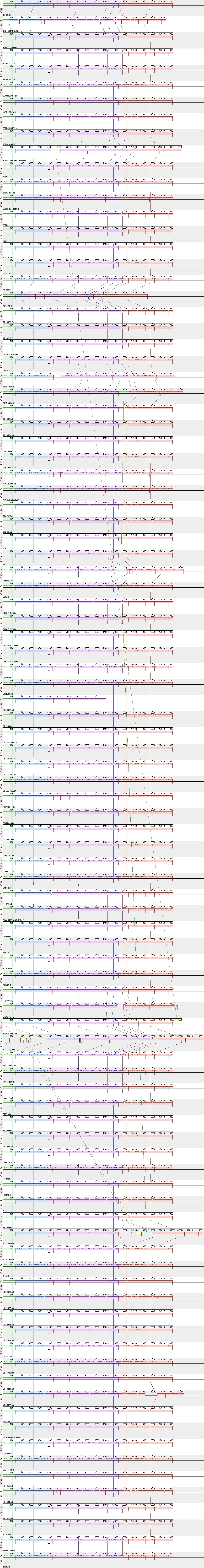

Supplement: Supplementary Figure 1 — Comparative analysis of the T6SS gene cluster from the A. baumannii genomes collected from Genbank. [file Image_1.jpeg]

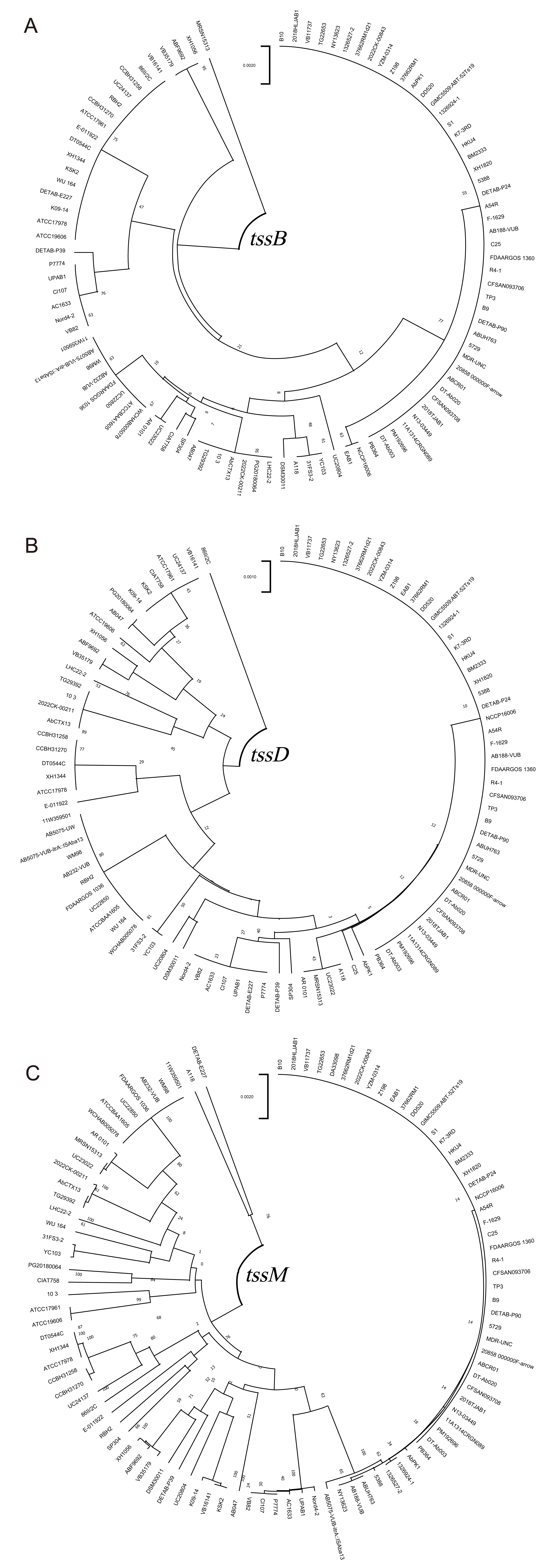

Supplement: Supplementary Figure 2 — Phylogeny of three T6SS core genes tssB (A), tssD (B) and tssM (C) from the A. baumannii genomes collected from Genbank were constructed by UPGMA model in MEGA, with the Bootstrap replications as 1000. [file Image_2.tif]

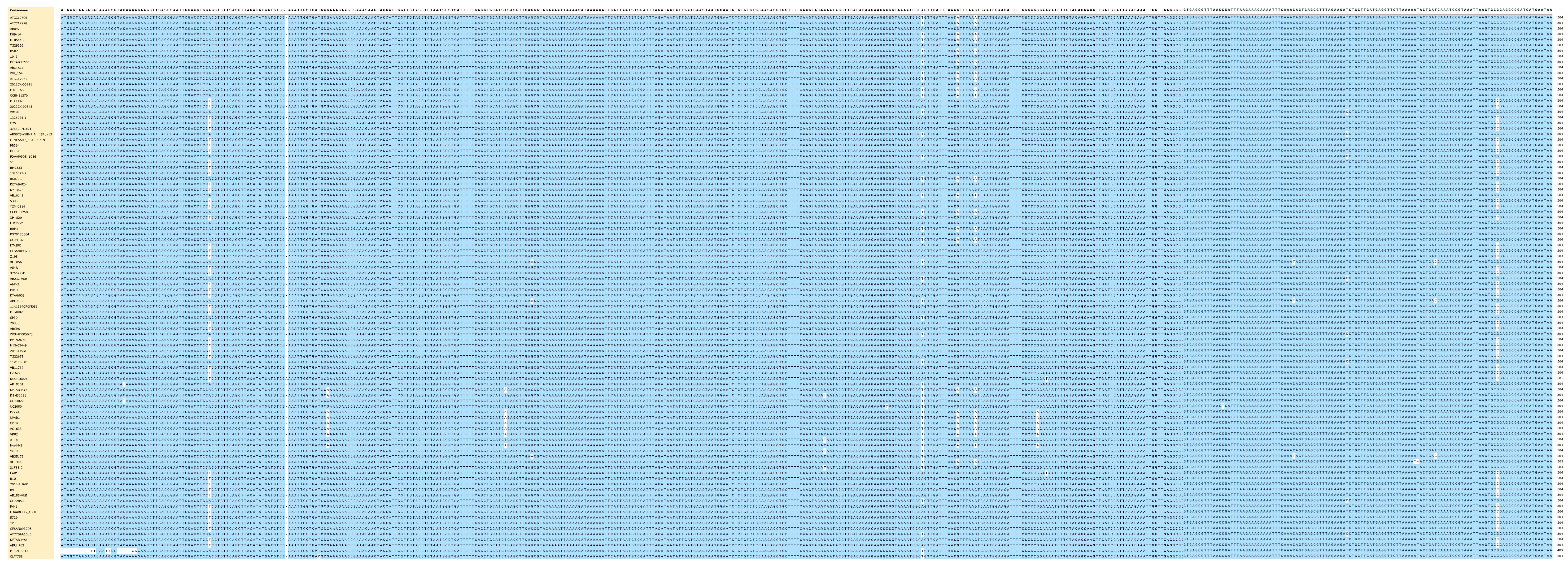

Supplement: Supplementary Figure 3 — The sequence alignment of tssB gene from the A. baumannii genomes collected from Genbank. [file Image_3.jpeg]

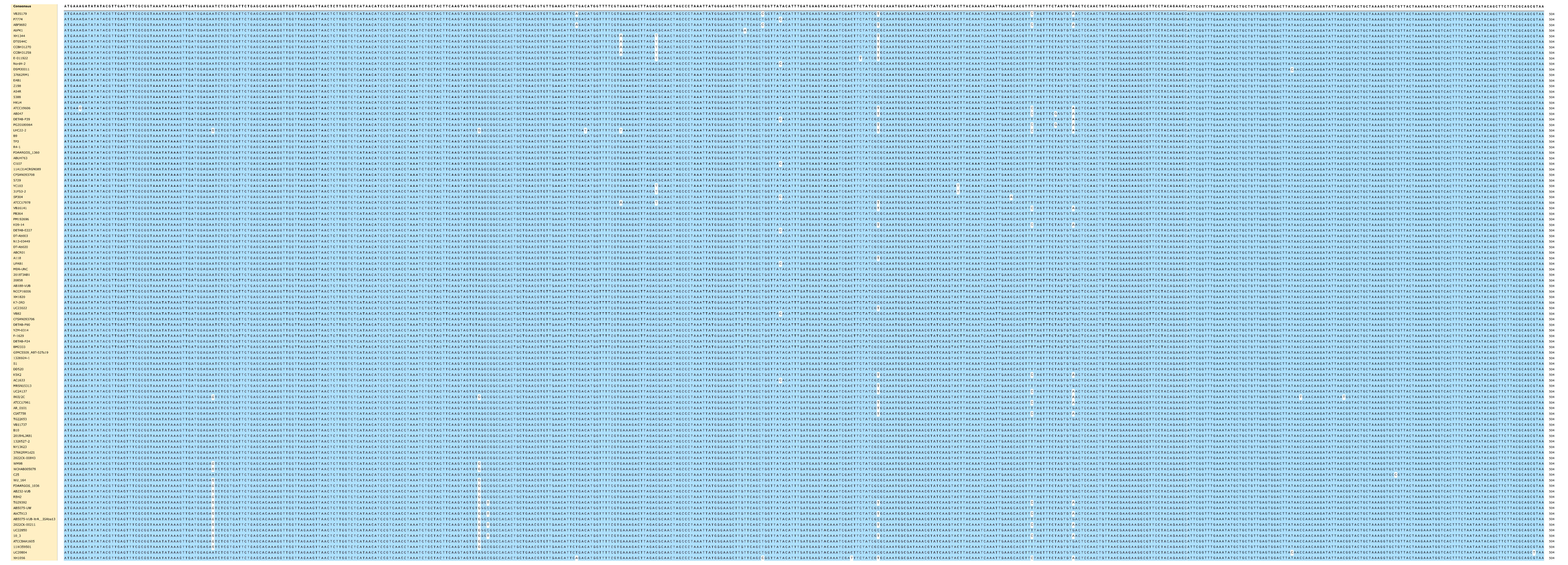

Supplement: Supplementary Figure 4 — The sequence alignment of tssD gene from the A. baumannii genomes collected from Genbank. [file Image_4.jpeg]

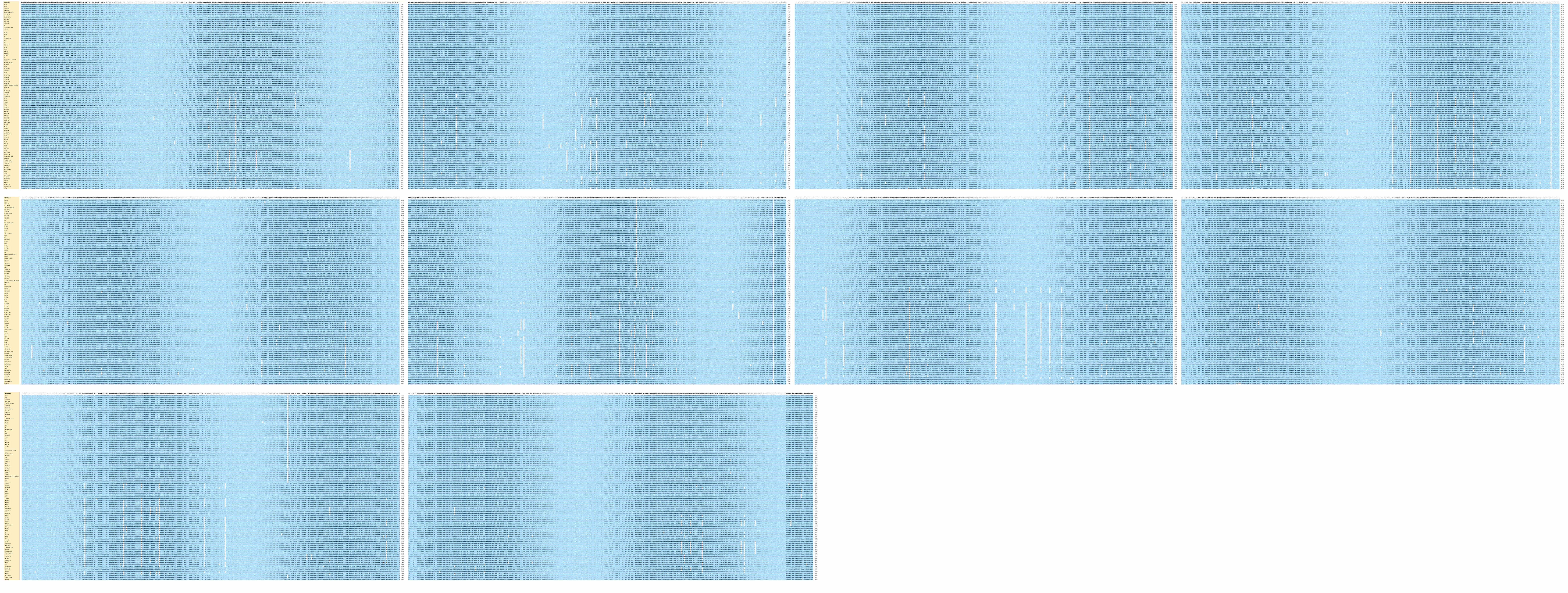

Supplement: Supplementary Figure 5 — The sequence alignment of tssM gene from the A. baumannii genomes collected from Genbank. [file Image_5.jpeg]

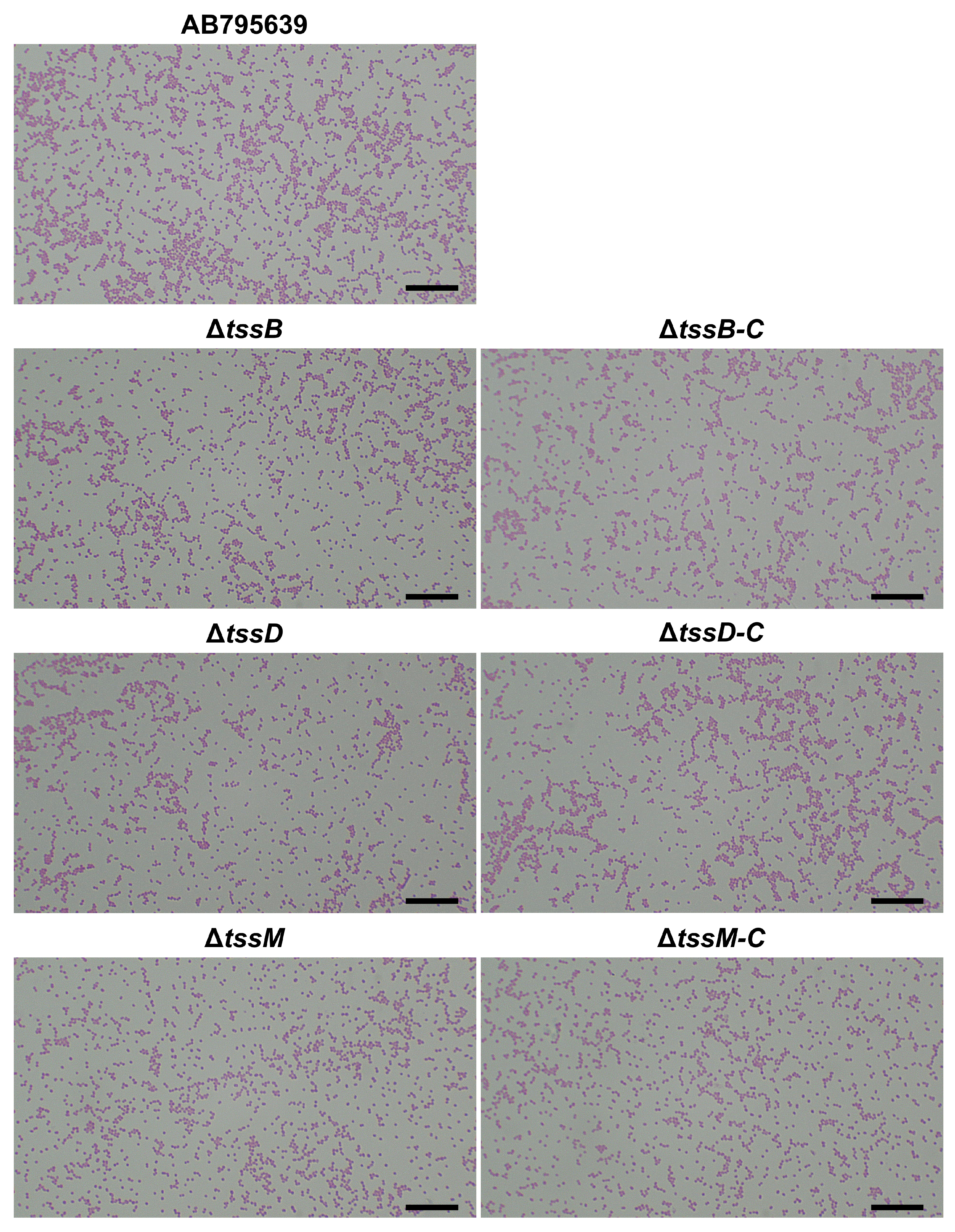

Supplement: Supplementary Figure 6 — Representative Gram-stain results of AB795639, the deletion and complementary mutants of three T6SS core genes. Scale bars: 100 pixels. [file Image_6.jpeg]
